# Do Human-Infecting *Yersinia enterocolitica* Isolates Exhibit Adaptive Phenotypes?

**DOI:** 10.3390/pathogens15050512

**Published:** 2026-05-11

**Authors:** Martine Denis, Emmanuelle Houard, Adiel Ouedraogo, Patricia Le Grandois, Carole Feurer, Clémence Bièche-Terrier, Cyril Savin, Christophe Soumet, Anne-Sophie Le Guern

**Affiliations:** 1 ANSES, Unit of Hygiene and Quality of Poultry and Pork Products (HQPAP), F-22440 Ploufragan, France; emmanuelle.houard@anses.fr (E.H.); ouedraogoadiel@gmail.com (A.O.); 2 ANSES, Unit of Antibiotics, Biocides, Residues, and Resistance (AB2R), F-35306 Fougères, France; patricia.legrandois@anses.fr (P.L.G.); christophe.soumet@anses.fr (C.S.); 3 IFIP, The French Pig and Pork Institute, Department of Fresh and Processed Meat, F-35850 Pacé, France; carole.feurer@ifip.asso.fr; 4 Idele, French Livestock Institute, Department of Carcass and Meat Quality, F-14310 Villers-Bocage, France; clemence.bieche@idele.fr; 5 Institut Pasteur, Université de Paris Cité, Yersinia Research Unit, National Reference Center for Plague & Other Yersiniosis, WHO Collaborating Centre for Plague Fra-140, F-75015 Paris, France; cyril.savin@pasteur.fr (C.S.); anne-sophie.le-guern@pasteur.fr (A.-S.L.G.)

**Keywords:** *Yersinia enterocolitica*, cold survival, motility, biofilm, biocide resistance, virulence

## Abstract

*Yersinia enterocolitica* strains of biotype 4 (BT4) are the most prevalent in human cases in France, followed by biotype 2 (BT2). We evaluated four BT4 porcine (P) isolates and four BT2 bovine (B) isolates for their ability to survive at 4 °C in culture broth and on meat, exhibit motility at 4 °C and 12 °C, adhere to stainless steel at 12 °C, resist five biocides, and infect human intestinal *Caco-2* cells. The objective was to determine whether animal isolates that genetically cluster with human (H) isolates (P+H+, B+H+) differ phenotypically from non-clustering isolates (P+H−, B+H−), based on core genome multi-locus sequence typing (cgMLST) using allelic distance thresholds of ≤5 for BT4 and ≤3 for BT2 isolates. No significant difference was observed for BT4 between P+H+ and P+H− isolates, nor for BT2 between B+H+ and B+H− isolates, for any test, except for motility. Porcine isolates clustering with human isolates (H+) exhibited a significantly reduced motility compared with non-clustering isolates (H−) (*p*-value < 0.05). In contrast, bovine isolates clustering with human isolates (H+) showed a significantly higher motility than non-clustering isolates (H−). Motility plays a role in the early stages of biofilm formation but is not directly involved in virulence, as *Y. enterocolitica* becomes non-motile at 37 °C. Animal isolates that did not cluster with human isolates displayed traits enabling their transmission along the food chain, suggesting potential low-level human exposure, asymptomatic carriage, or links to unreported infections.

## 1. Introduction

*Yersinia enterocolitica* is the fourth most common bacterial cause of human diarrhea in Europe [1], after *Campylobacter*, *Salmonella*, and Shiga toxin-producing *Escherichia coli* (STEC) infections. Although reporting of human yersiniosis cases and monitoring of *Y. enterocolitica* in food and animals are mandatory in most European Union Member States, yersiniosis is not a notifiable disease in France. Nevertheless, *Y. enterocolitica* is covered by Directive 2003/99/EC on the monitoring of zoonoses and zoonotic agents. As a result, official surveillance data in France do not provide a reliable estimate of the true annual number of foodborne yersiniosis cases, which has nevertheless been estimated at approximately 23,000 cases per year for the period 2008–2013 [2].

*Y. enterocolitica* is subdivided into six biotypes (BT), of which BT1A is considered non-pathogenic, whereas BT1B, BT2, BT3, BT4, and BT5 are pathogenic. In France, among *Y. enterocolitica* from human cases, BT4 is the most frequently detected (71.1%), followed by BT2 (25.4%) and BT3 (2%) [3]. An analysis of the origins of animal *Y. enterocolitica* isolates collected by the French Reference Centre for plague and other yersiniosis between 1931 and 2013 [3] showed that BT4 isolates mainly originated from the pig production sector, accounting for 87.9% of BT4 isolates. This finding is supported by surveys conducted in French pig slaughterhouses [4,5] and at retail on pig products [6]. In contrast, only 3.5% of BT2 isolates originated from pigs, while the majority (54.7%) were associated with the ruminant sector, predominantly cattle [3].

A recent study on French *Y. enterocolitica* isolates identified genetic clusters in which porcine and bovine isolates were grouped with human isolates, by core genome multilocus sequence typing (cgMLST) using allelic distance thresholds of ≤5 for BT4 and ≤3 for BT2 isolates. This study, thereby, confirmed pigs as the probable source of BT4 and cattle as the probable source of BT2 [7]. In contrast, some BT4 porcine and BT2 bovine isolates did not cluster with human isolates, suggesting that some animal *Y. enterocolitica* isolates may be less likely to be transmitted through the food chain and/or to infect humans.

For *Y. enterocolitica* isolates to infect humans, they must be able to pass through the entire food chain—from the animal reservoir to the consumer. This requires the ability to withstand various stresses, including exposure to biocides used in livestock farming and the food industry, as well as survival at refrigeration and food-processing conditions. *Yersinia enterocolitica* is known to tolerate low temperatures, and this psychotropic ability allows it to multiply in foods stored under refrigeration [8].

Moreover, *Y. enterocolitica* can form biofilms, which enhances its persistence in the environment and increases its resistance to antimicrobial agents. Motility and biofilm formation in *Y. enterocolitica* are influenced by environmental factors such as temperature, calcium concentration, and the presence of the virulence plasmid pYV [9]. In addition, animal-derived *Y. enterocolitica* isolates must remain viable on food matrices and retain their pathogenic potential to infect humans. Numerous virulence determinants, both plasmid- and chromosome-encoded, play a key role in the infection process [10].

Using in vitro assays, we evaluated the ability of eight *Y. enterocolitica* animal isolates to survive at 4 °C in culture broth and on meat, to exhibit motility at 4 °C and 12 °C, to adhere to stainless steel at 12 °C, to resist five biocides, and to infect human intestinal *Caco-2* cells. These experiments aimed to determine whether porcine (P) and bovine (B) isolates that genetically cluster with human (H+) isolates (P+H+ and B+H+), as defined by cgMLST [11], differed phenotypically from those that do not cluster with human isolates (P+H− and B+H−).

## 2. Materials and Methods

### 2.1. Yersinia enterocolitica Isolates

In the study of Savin et al. [7], genomes of porcine *Yersinia enterocolitica* BT4 isolates and bovine *Y. enterocolitica* BT2 isolates were compared with genomes of human isolates by cgMLST. Several clusters were identified, in which genomes of animal isolates were closely related to the genomes of human isolates. The affiliation of a porcine (P) or bovine (B) strain in a cgMLST cluster containing at least one human (H) isolate was determined according to the allelic distance (AD) between the genomes. This AD was ≤ than 5 alleles for BT4, and ≤ than 3 alleles for BT2 [11].

For in vitro testing, we selected eight isolates of *Y. enterocolitica* of animal origin, which were distributed into four groups, P+H+, P+H−, B+H+, and B+H−, depending on whether these isolates clustered or not with human isolates. Each group contained two isolates, and the allelic distance (AD) to human strains is indicated in Table 1. Genomic sequencing confirmed that all eight isolates harbored the pYV virulence plasmid. The genomes of these isolates are freely available in the database https://bigsdb.pasteur.fr/yersinia (accessed on 30 January 2026). Id numbers are 14281, 14284, 13663, 13746, 14267, 14272, 13674, and 13723 for EV-C1-B4-P-016, EV-C1-B4-P-013, EV-C1-B2-B-017, EV-C1-B2-B-045, EV-C1-B4-P-005, EV-C1-B4-P-025, EV-C1-B2-B-030, and EV-C1-B2-B-032, respectively.

### 2.2. Survival of Y. enterocolitica in Cold Conditions

#### 2.2.1. In Culture Broth

First, the survival of *Y. enterocolitica* isolates was tested at 4 ± 2 °C in Brain Heart Infusion (BHI; Biokar , Allonne, France). For each isolate, a colony grown on Plate Count Agar (PCA; Biomérieux, Marcy l’Etoile, France) at 30 °C for 24 h was used to inoculate 5 mL of BHI using a one-µL loop. After incubation at 30 °C for 18 h, the culture was serially diluted (1:10) in BHI up to 10^−4^ for a target concentration of approximately 5 Log_10_ CFU/mL. This last dilution was divided into five tubes of two mL and stored at 4 ± 2 °C. Enumeration of *Y. enterocolitica* (CFU/mL) was performed on PCA on the day of inoculation (D0) and after 4 (D04), 6 (D06), 8 (D08), and 11 (D11) days of storage. Colonies were counted after 24 h at 30 °C of incubation of the plates. The assay was performed in triplicate at separate times. The absence of *Y. enterocolitica* growth in non-inoculated BHI was verified by streaking BHI on cefsulodine—irgasine—novobiocine (CIN) agar plates (Thermofisher Diagnostic SAS, Dardilly, France). Plates were incubated at 30 °C for 24 h.

#### 2.2.2. On Meat

Secondly, the survival of *Y. enterocolitica* isolates was tested on pork ham for BT4 porcine isolates and on beef steak for BT2 bovine isolates. For each isolate, a colony grown on PCA at 30 °C for 24 h was used to inoculate 5 mL of BHI using a one-µL loop. After incubation at 30 °C for 18 h, the culture was serially diluted (1:10) in Tryptone Salt (TS; Biomérieux) up to 10^−2^. Slices of ham and raw beef steaks were purchased from a butcher on the day of inoculation. For each isolate, seven 25 cm^2^ squares of ham or steak were placed individually in a sterile Petri dish and inoculated with 50 µL of the dilution 10^−2^ to achieve a target concentration of approximately 4–4.5 Log_10_ CFU/ cm^2^. After spreading the inoculum, squares were placed at 4 ± 2 °C. The experiment was conducted over 10 days to simulate the time interval between meat (ham or steak) leaving the processing facility and its storage in the consumer’s refrigerator, under continuous cold-chain conditions. Enumeration of *Y. enterocolitica* was performed on the day of the inoculation (D0), and after 1 (D01), 2 (D02), 3 (D03), 6 (D06), 8 (D08), and 10 (D10) days of storage after inoculation. At each time point, one square was stomached in 20 mL of TS and used for enumeration on CIN plates. Typical colonies were counted after 24 h of incubation at 30 °C on the plates, and results were expressed in Log_10_CFU per cm^2^. The assay was performed in triplicate at separate times. In each test, an additional square of ham or steak was analyzed according to standard ISO 10273:2017 [12] to confirm the absence of *Y. enterocolitica* prior to inoculation.

### 2.3. Swimming Motility of Y. enterocolitica 

The swimming motility of the isolates was assessed based on their ability to move in a semi-solid BHI medium containing 0.27% agar. For each isolate, we prepared two 12-well microplates filled with semi-solid BHI for the motility test at 4 ± 2 °C, and two additional 12-well microplates for the motility test at 12 ± 2 °C. For each isolate, from a culture at 10^6^ CFU/mL (obtained as described previously), we realized a 1:5 serial dilution on eight consecutive BHI tubes. Then, 20 μL of each dilution was deposited on semi-solid BHI in the microplate, with 3 wells per column corresponding to each dilution.

One set of microplates was incubated at 12 ± 2 °C (average temperatures corresponding to conditions encountered during daily activity in a slaughterhouse (around 20 °C) and in a cutting plant (up to 8 °C max)), while the other set was incubated at 4 ± 2 °C (corresponding to refrigeration temperature). Each day over a 10-day period, no growth (NG), presence of colonies restricted to the point of deposition (D), or migration halo (H) was scored for each well in order to monitor motility dynamics. The experiment was performed in triplicate, resulting in a total of 720 scores per isolate over the 10 days (two 12-well microplates × three replicates × 10 days).

### 2.4. Ability to Adhere on Stainless Steel Coupons

The stainless-steel (Maplaqueinox, Tournan en Brie, France) coupons measured 2.5 cm × 2.5 cm and had a surface roughness (Ra) of 0.6–1.2 µm, consistent with normal surface abrasion. The coupons were first degreased using a 50% (*v*/*v*) acetone/ethanol solution with Kimwipes-type wipes. They were then immersed in a 2% (*v*/*v*) solution of RBS 35 (R. Borghgraef S.a, Bruxelles, Belgique) in ultrapure water at 50 °C and gently agitated with a magnetic stir bar for 10 min in a glass crystallizer. The coupons were subsequently rinsed for 5 min in ultrapure water at 50 °C, followed by a further 5 min rinse in ultrapure water at room temperature in the glass crystallizer. Once cleaned, the coupons were handled using sterile tweezers.

For the assay, a stainless-steel coupon was placed in petri dishes and exposed to an average inoculum of 3 × 10^9^ *Y. enterocolitica* cells on the surface for 6 h at 12 ± 2 °C, a duration and an average temperature approaching daily activity conditions in a cutting plant. Non-adherent bacterial cells were removed by rinsing the coupon five times with TS. The coupon was then placed in a vial containing 10 mL of TS. The vial was placed in an ultrasonic bath for 2 min to detach adherent cells. The resulting suspension was serially diluted (1:10) in TS and plated on PCA for the enumeration of cells that had adhered to the stainless steel surface. Plates were incubated at 30 °C for 24 h. Results were expressed as the number of adherent cells on the stainless-steel coupon, and expressed in percentage of adhesion (number of adherent cells on stainless-steel coupon × 100/number of cells in contact with stainless-steel coupon). The test was performed in triplicate at separate times.

### 2.5. Susceptibility to Biocides

The susceptibility of the isolates was assessed by determining the Minimum Inhibitory Concentration (MIC) using the microdilution method against five biocides. The selected biocides are active substances belonging to different families of chemical compounds and are components of commercial biocidal products used for cleaning and disinfecting in livestock farming and processing plants. These include benzalkonium chloride (BC) and Didecyl Dimethyl Ammonium Chloride (DDAC) from the quaternary ammonium family, N-(3-aminopropyl)-N-dodecylpropane-1,3-diamine (AMPD) from the amines, peracetic acid (PA) from the peroxides, and Sodium Hypochlorite (NaOCl) from the chlorine-based compounds.

The *Y. enterocolitica* isolates were inoculated onto nutrient agar and incubated at 30 °C for 18 h. Then, three to four colonies were transferred into 10 mL of Tryptic Soy Broth (TSB) and incubated at 30 °C for 18 h. The bacterial suspensions were standardized by measuring the optical density at 600 nm in TSB to obtain a final concentration of 10^5^ CFU/mL.

Serial dilutions of the biocides were freshly prepared in tap water to obtain the following concentrations: BC (40–30–20–15–10–5 µg/mL), DDAC (20–10–5–2.5–1.25–0.625 µg/mL), AMPD (400–300–200–150–100–50 µg/mL), PA (10,000–5000–2500–1250–625–312–156–78–39–19 µg/mL), and NaOCl (640–320–160–80–40–20 µg/mL).

Biocide solutions were dispensed in a 96-well microplate with 20 µL added to each well in a column of eight wells. Bacterial suspensions adjusted to 10^5^ CFU/ mL were then added to each well at 180 µL per well. Two controls were included: a growth control, consisting of 20 µL of bacterial suspension added to 180 µL of broth medium (TSB without biocides), and a sterility control, consisting of 20 µL of tap water (instead of biocide) added to 180 µL of broth medium (TSB without bacteria).

After 48 h of incubation at 30 °C, the presence or absence of turbidity in each well was noted visually to determine the MIC value for each strain. The MIC was defined as the lowest concentration at which no visible bacterial growth was observed. For AMPD, measurements were performed using a FluoStar microplate reader (BMG Labtec, Champigny-sur-Marne, France), as the TSB-AMPD mixture becomes cloudy and prevents reliable visual determination of growth. Two independent experiments were conducted on different days with three technical replicates per experiment.

### 2.6. Adhesion and Invasion on Caco-2 Cells

The pathogenicity of *Y. enterocolitica* was assessed using adhesion and invasion assays performed on human intestinal *Caco-2* cells, as previously described [13]. *Caco-2* cells (ECACC 86010202) were obtained from the European Collection of Cell Culture (ECACC, Salisbury, UK). Overnight cultures of *Y. enterocolitica* were grown at 30 °C in a BHI medium. *Caco-2* cells were cultured at 37 °C in an atmosphere containing 5% CO_2_. Cells were maintained in Dulbecco’s Modified Eagle Medium (DMEM; GIBCO Thermofisher Scientific, Illkirch, France) supplemented with 10% fetal bovine serum (FBS; GIBCO Thermofisher Scientific, Illkirch, France), 1% non-essential amino acids (NEAA; GIBCO Thermofisher Scientific), and 20 mM HEPES (4-(2-hydroxyethyl)-1-piperazineethanesulfonic acid; GIBCO Thermofisher Scientific, Illkirch, France). Prior to infection, 10^5^ *Caco-2* cells were seeded into individual wells of 24-well tissue culture plates and allowed to grow for 48 h at 37 °C.

Overnight cultures of *Y. enterocolitica* were centrifuged for 15 min at 4500 rpm. The pellet was then resuspended in 5 mL of DMEM supplemented with 10% FBS. The optical density was measured at 600 nm to obtain a final bacterial concentration of 2.10^7^ CFU/mL. For infection, approximately 2.10^7^ bacteria were added to 2.10^5^ *Caco-2* cells, corresponding to a multiplicity of infection (MOI) of 100. The plates were incubated at 37 °C in a 5% CO_2_ atmosphere. Each plate included a well containing only *Caco-2* cells, a well containing bacteria only (without *Caco-2* cells), and two positive controls consisting of previously tested BT4 (Y09AL405) and BT2 (Y10FM0700) strains [13].

Three hours post-infection, the cells were washed with phosphate-buffered saline (PBS). The total number of adherent bacteria was determined by lysing the cells with 0.1% Triton X-100, followed by enumeration on PCA plates after a serial tenfold dilution in a TS medium. Bacterial internalization was assessed by adding gentamicin (100 µg per well) to kill extracellular bacteria, followed by an additional 2 h incubation at 37 °C in a 5% CO_2_ atmosphere. The number of intracellular bacteria was then determined by enumeration on PCA plates after a serial tenfold dilution in the TS medium.

For each isolate, adhesion and invasion efficiencies were calculated as the number of recovered CFU relative to the total number of bacteria initially added to the *Caco-2* cells. For each strain, the results were the mean of three independent determinations.

### 2.7. Statistical Analyses

All the statistical analyses were performed in R version 4.5.2. Comparisons of means were conducted using the Wilcoxon test, except for the motility assay, which was analyzed using Pearson’s Chi-squared test. A Bonferroni correction was applied, as only two biotypes (BT4 and BT2) and two strains per group (P+H+, P+H−, B+H+, and B+H−) were considered. Differences were considered statistically significant when the *p*-value was < than 0.05. All raw data are available in the Supplementary data file.

## 3. Results

### 3.1. Growth of Y. enterocolitica at Low Temperature

**Figure 1:**
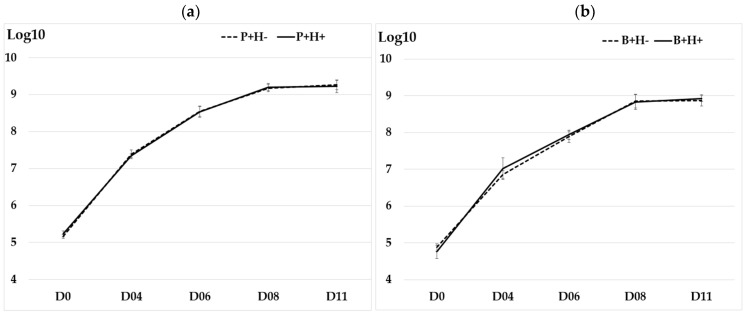

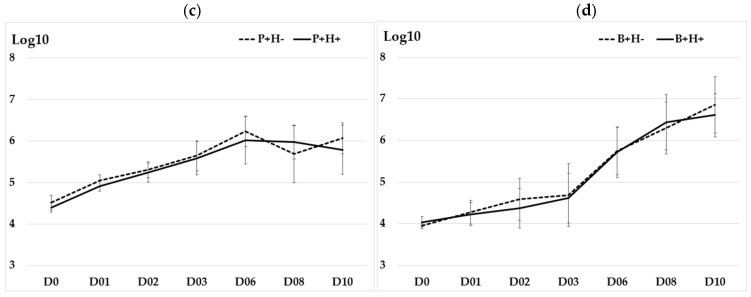
Growth curves of the two *Y. enterocolitica* isolates per group (P+H−, P+H+, B+H−, and B+H+) at 4 °C over time in days (D). (**a**) growth curve of BT4 porcine isolates in Brain Heart Infusion in Log_10_CFU per ml; (**b**) growth curve of BT2 bovine isolates in Brain Heart Infusion in Log_10_CFU per ml; (**c**) growth curve of BT4 porcine isolates on ham in Log_10_ CFU per cm^2^; and (**d**) growth curve of BT2 bovine isolates on beef steak in Log_10_CFU per cm^2^. The assays were performed in triplicate at separate times. The vertical bars for each mean correspond to the standard deviation.

All isolates of *Y. enterocolitica* multiplied in BHI placed at 4 °C, reaching or exceeding 10^9^ cells by day D11 (Figure 1a,b). No significant difference was observed between P+H+ and P+H− isolates (*p*-value = 0.968) or between B+H+ and B+H− isolates (*p*-value = 0.925). All porcine isolates grew on ham at 4 °C (Figure 1c), with no significant difference between P+H+ and P+H− isolates (*p*-value = 0.258). Similarly, all bovine isolates grew on beef steak at 4 °C (Figure 1d) with no significant difference between B+H+ and B+H− isolates (*p*-value = 0.701). The means and standard deviation (SD) of the growth curves in BHI over 11 days and on meat over 10 days for isolates from the four groups (P+H−, P+H+, B+H−, and B+H+) are provided in Table S1.

However, differences in growth dynamics were observed in meat between BT4 porcine isolates and BT2 bovine isolates. This variation may be related to the matrices and their associated microflora, as both biotypes exhibited similar growth behavior in BHI.

### 3.2. Variability in Swimming Motility Among Isolates

*Y. enterocolitica*, whatever its origin (porcine or bovine), was able to multiply at 4 °C and 12 °C and swim in a semi-solid medium, forming a migration halo (Table 2a–d). However, significant differences (*p*-value < 0.05) were observed between porcine and bovine isolates, with a higher number of migration halos for bovine isolates. Motility of the isolates was also significantly greater at 12 °C than at 4 °C (*p*-value < 0.05).

Motility differed significantly between P+H+ and P+H− isolates at both 4 °C and 12 °C, and between B+H+ and B+H− isolates only at 12 °C (Table 2a–d). Specifically, the number of migration halos at 4 °C and at 12 °C was higher for P+H− than for P+H+, whereas at 12 °C, it was lower for B+H− than for B+H+. These results suggest that BT4 porcine isolates, which genetically cluster with human isolates (H+), exhibit lower motility than isolates not genetically clustered with human isolates (H-), whereas the opposite trend is observed for BT2 bovine strains.

### 3.3. No Significant Differences in Stainless Steel Adhesion Among Isolates

Under our experimental conditions (i.e., from an average inoculum of 3 × 10^9^ *Y. enterocolitica* cells, after 6 h of contact at 12 °C on stainless steel), an average of 4 × 10^7^ adherent cells were recovered from the stainless steel, corresponding to adhesion rates ranging from 0.52% to 2.13% for porcine isolates and from 0.90% to 2.30% for bovine isolates. No significant difference was observed between BT4 isolates and BT2 isolates in their ability to adhere to stainless steel surfaces (*p*-value = 0.386). Moreover, no significant difference was found between P+H− and P+H+ isolates (*p*-value = 0.484), nor between B+H− and B+H+ isolates (*p*-value = 0.240).

### 3.4. MIC Profiles of *Y. enterocolitica* Isolates for Tested Biocides

**Figure 2:**
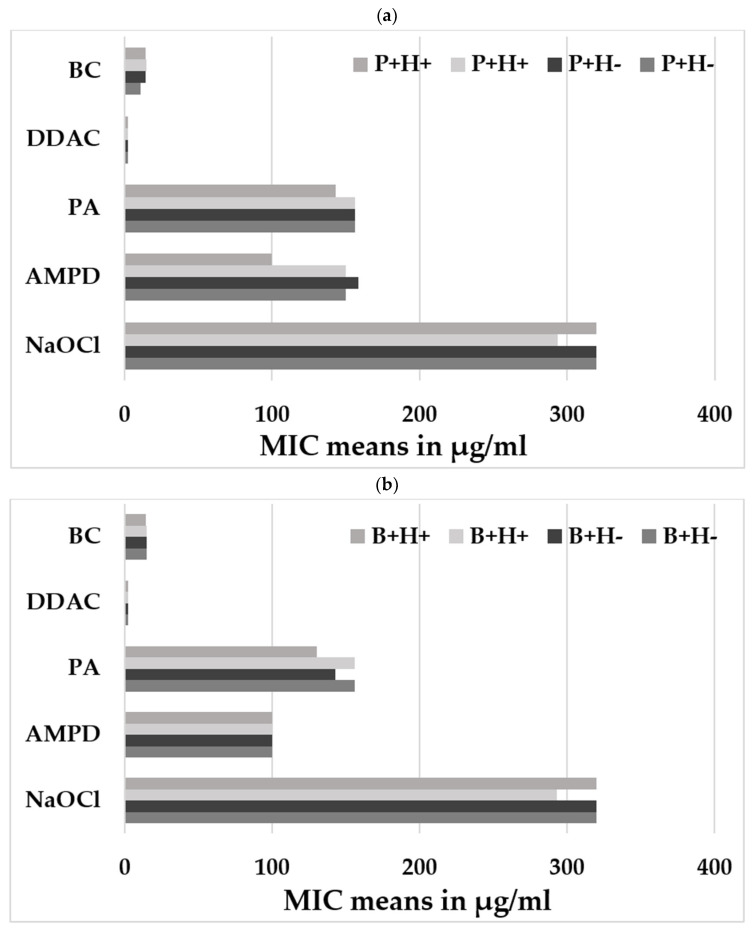
Susceptibility to the five tested biocides: (**a**) means of MIC for the isolates P+H−, P+H+; (**b**) means of MIC for the isolates B+H− and B+H+. Benzalkonium chloride (BC) and Didecyl Dimethyl Ammonium Chloride (DDAC) from the quaternary ammonium family, peracetic acid (PA) from the peroxides, N-(3-aminopropyl)-N-dodecylpropane-1,3-diamine (AMPD) from the amines, and Sodium Hypochlorite (NaOCl) from the oxidants. Two independent experiments were conducted on different days with three technical replicates per experiment.

Significantly higher MIC values were observed for the amino compound (AMPD), reaching 150 µg/mL for porcine isolates (except one isolate at 100 µg/mL) (Figure 2a), compared to bovine isolates (*p*-value = 0.0023) (Figure 2b). However, no significant differences (*p*-values > than 0.05) were observed in MIC values for any of the five biocides tested between the P+H− and P+H+ isolates, nor between the B+H− and B+H+ isolates (Figure 2a,b).

### 3.5. Behavior of the Isolates on Caco-2 Cells

BT4 porcine isolates were significantly more adherent (*p*-value = 0.001) to *Caco-2* cells than the BT2 bovine isolates. However, no significant difference was observed between the two biotypes in terms of invasion capacity to *Caco-2* cells (*p*-value = 0.599).

There was no significant difference between P+H+ and P+H− isolates in either adhesion (72.5% +/−13.7 vs. 81.6% +/−23.0) (*p* = 0.699) or invasion of *Caco-2* cells (2.7% +/−0.5 vs. 3.6% +/−1.1) (*p* = 0.240). Similarly, within BT2 isolates, no significant difference was observed between the B+H+ and B+H− isolates, in either adhesion (25.1% +/−4.0 vs. 21.8% +/−3.6) (*p* = 0.818) or invasion of *Caco-2* cells (3.6%+/−0.9 vs. 2.9%+/−1.3) (*p* = 0.484).

## 4. Discussion

In this study, we evaluated the ability of eight pathogenic animal *Y. enterocolitica* isolates to withstand environmental stresses (including low temperatures and exposure to disinfectants), to adhere to stainless steel surfaces, and to infect human intestinal cells, according to their genetic relatedness to human isolates. However, our results should be interpreted with caution as only two strains per group were analyzed.

All isolates tested in this study were able to survive and multiply in culture broth and on meat at low temperature (4 °C), and no significant differences were observed between the P+H+ and P+H− isolates, nor between the B+H+ and B+H− isolates. Enteropathogenic *Y. enterocolitica* are psychotropic bacteria capable of growing at temperatures close to 0 °C, which poses major challenges for the modern food industry [14]. It has been reported that cold acclimation at the transcriptional level involves the transient induction followed by the effective repression of cold shock protein (Csp) genes [8]. In addition, changes in membrane fluidity and motility play an important role in the cold stress response of *Y. enterocolitica* [15].

In our study, we observed that swimming motility was temperature-dependent, being significantly lower at 4 °C than at 12 °C. It has been reported that during cold shock, membrane fluidity decreases [15], which negatively affects the motility of *Y. enterocolitica* isolates. Our results are consistent with previous observations reported for *Y. enterocolitica* [16].

We also highlighted that motility was higher in BT2 bovine isolates than in BT4 porcine isolates. In some BT2 isolates, an additional flagellar gene cluster (Flag-2), which is absent or differently expressed in other biotypes, has been described [17]. In *Y. enterocolitica* BT2, this Flag-2 cluster encodes flagellar components expressed at low temperatures (approximately 20 °C), and is associated with modified motility. We verified the presence of the Flag-2 cluster by performing a BLASTn analysis of the nucleotide sequence (accession number AM600695, [17]) against the eight genomes included in this study. The gene cluster Flag-2 was detected in all BT2 strains with 99.8% identity, whereas the BT4 strains exhibited lower identity (94%), which may support our observations.

We observed significant differences in motility between the P+H+ and P+H− isolates, as well as between the B+H+ and B+H− isolates. Our results suggest that porcine isolates genetically clustering with human isolates (H+) exhibited lower swimming motility than isolates not genetically clustering with human isolates (H−), whereas bovine isolates genetically clustering with human isolates (H+) exhibited, conversely, higher swimming motility than isolates not genetically clustering with human isolates (H−). Swimming motility is flagellum-dependent [16]. Flagella are essential for bacterial motility and surface colonization and are also involved in biofilm formation and bacterial pathogenesis [18].

Mutations were identified in the two B+H− isolates compared to the two B+H+ isolates, involving a different allele of the *fliM* gene, based on the genome analysis previously reported by Savin et al. [7]. The FliM protein is a key component of the bacterial flagellar motor switch complex [19], which controls the direction of flagellar rotation [20] and enables bacteria to navigate their environment *via* chemotaxis [21]. Mutations in *fliM* can result in rotation locked in a single direction, defective chemotaxis, or impaired flagellar assembly [22]. The mutations observed in the *fliM* gene of the B+H− strains support the finding that these strains are less motile. Flagellar mutants should be tested to confirm these observations.

We also identified that the P+H+ and P+H− isolates carried different alleles of three flagellin-related genes: *fliC*, *fliF*, and *fliO*. Variations in these alleles, including mutations, deletions, or gene absence, may result in non-flagellated bacteria (unable to swim), shortened flagella, or defective motility [23,24]. However, in our study, these allele differences were detected only in the isolates selected for in vitro testing, suggesting that this finding cannot be generalized to all P+H+ and P+H− isolates identified in our previous study [7].

All of our strains were able to adhere to the stainless steel, as evidenced by the recovery of an average of 4.10^7^ adherent cells from this surface after sonication. No differences were observed among isolates regardless of biotype or group affiliation. Wide variability in biofilm formation among poultry-derived *Y. enterocolitica* isolates was previously reported [25], where some isolates did not form biofilms, others produced moderate or strong biofilms, and a minority exhibited very high biofilm production. It has been shown that the intensity of biofilm production depended on the colony morphology of *Y. enterocolitica* on solid nutrient media [26]. More recently, it has been demonstrated that biofilm formation by *Y. enterocolitica* has been demonstrated to be influenced by environmental factors such as temperature, calcium concentration, and the presence or absence of the virulence plasmid pYV [9]. In our study, all isolates were tested at 12 °C, hosted the pYV plasmid, and exhibited no differences in colony morphology under stereomicroscopic observations. Consequently, their genetic proximity to human isolates does not appear to be related to their ability to adhere to stainless steel surfaces.

The biofilm lifestyle enables bacterial colonies to persist in an environment while being protected from various stresses, including antibacterial agents, even in the absence of active proliferation. *Y. enterocolitica* isolates exposed to sublethal concentrations of biocides may develop resistance to these agents [27]. The results of this previous study [27] also suggest that concentrations of peracetic acid (PA) and benzalkonium chloride (BC) close to the minimum inhibitory concentrations (MICs) may promote the release of viable bacteria from biofilms, posing a potential public health risk. The higher susceptibility to AMPD observed in biotype BT2 compared to BT4 may be explained by differences in biofilm-forming capacity. Indeed, BT4 strains have been shown to form stronger and more persistent biofilms under conditions mimicking food-processing environments, which may contribute to their enhanced tolerance to biocides [28]. However, no differences in adhesion to stainless steel were observed between the strains analyzed in this study. In addition, within the same biotype, isolates genetically clustering (H+) or not (H−) with human isolates exhibited similar or identical biocide susceptibility profiles, suggesting that they may share similar capacities to withstand biocide treatments used in food industry settings.

As previously described [13], *Y. enterocolitica* BT2 isolates exhibited a lower capacity to adhere to *Caco-2* cells compared to BT4 isolates, while no significant difference was observed between the biotypes in their ability to invade *Caco-2* cells. Colonization properties of *Y. enterocolitica* have been shown to be mainly bioserotype-specific [29]. In our study, no significant differences were observed between the P+H+ and P+H− isolates or between the B+H+ and B+H− isolates, either in the adhesion to or invasion of *Caco-2* cells. This lack of difference may be explained by the repression of flagellar synthesis and the absence of motility at 37 °C in *Y. enterocolitica*. Overall, our results suggest that animal isolates not genetically clustering with human isolates have a similar capacity to infect human cells as those closely related to human isolates.

## 5. Conclusions

Our study primarily revealed differences in motility at 4 °C and 12 °C between BT2 and BT4 isolates, as well as between animal isolates clustering or not with human isolates. Although motility plays an important role during the early stages of biofilm formation, it does not appear to contribute to the adhesion and invasion of human intestinal cells, as *Y. enterocolitica* becomes non-motile at 37 °C. The observation that certain animal isolates, which possess the traits enabling them to cross the food chain, are not genetically clustered with human isolates may suggest that these isolates have either not been ingested by humans, do not induce symptoms in humans due to low infection doses, or are associated with unreported human infections. Indeed, in France, yersiniosis is not a notifiable disease. Our findings should be interpreted with caution, as only two strains per group were evaluated. Additional strains should be tested to confirm the robustness of these results.

## Figures and Tables

**Table 1:** Characteristics of the eight isolates selected for in vitro testing.

Groups	Strains	Biotype (BT)	AD with Human Strains
P+H+	EV-C1-B4-P-016	BT4	4
porcine strains clustered with human strains	EV-C1-B4-P-013	BT4	3
P+H−	EV-C1-B4-P-005	BT4	>9
porcine strains non-clustered with human strains	EV-C1-B4-P-025	BT4	>9
B+H+	EV-C1-B2-B-017	BT2	3
bovine strains clustered with human strains	EV-C1-B2-B-045	BT2	3
B+H−	EV-C1-B2-B-030	BT2	>6
bovine strains non-clustered with human strains	EV-C1-B2-B-032	BT2	>6

**Table 2:** Number of NG (no growth), D (colonies at the point of deposition), and H (halo of migration) for the evaluation of the motility of the isolates at 4 °C and 12 °C over 10 days with Pearson’s Chi-squared test and Bonferroni correction. A total of 1440 scores per group corresponds to 24 scores (2 × 12 well-microplates) recorded on 10 days, three times, and including the two strains per group, P+H+, P+H−, B+H+, and B+H−. The data are obtained from three replicates per dilution over 10 days, and the assay was repeated three times at different time points. (**a**) motility of porcine strains at 4°C, (**b**) motility of porcine strains at 12°C, (**c**) motility of bovine strains at 4°C, (**d**) motility of bovine strains at 14°C.

(a)							
Motility	Number of Score				Pearson’s Chi-Squared Test	Bonferroni Correction	
at 4 °C	NG	D	H	Total	*p*-Value	Adjusted *p*-Value	
P+H−	914	451	75	1440	*p* ≈ 1.12 × 10^−17^	NG vs. D	0.0282
P+H+	1029	409	2	1440		NG vs. M	3.57 × 10^−17^
Total	1943	860	77	2880		D vs. M	1.96 × 10^−13^
**(b)**							
**Motility**	**Number of Score**				**Pearson’s Chi-Squared Test**	**Bonferroni Correction**	
**at 12 °C**	**NG**	**D**	**H**	**Total**	***p*** **-Value**	**Adjusted** ***p*****-Value**	
P+H−	315	299	826	1440	*p* ≈ 2.3 × 10^−32^	NG vs. D	7.2 × 10^−12^
P+H+	360	593	487	1440		NG vs. M	1.1 × 10^−22^
Total	675	892	1313	2880		D vs. M	2.0 × 10^−27^
**(c)**							
**Motility**	**Number of Score**				**Pearson’s Chi-Squared Test**	**Bonferroni Correction**	
**at 4 °C**	**NG**	**D**	**H**	**Total**	***p*** **-Value**	**Adjusted** ***p*****-Value**	
B+H−	853	294	293	1440	*p* ≈ 0.977	NG vs. D	1.000
B+H+	855	295	290	1440		NG vs. M	1.000
Total	1708	589	583	2880		D vs. M	1.000
**(d)**							
**Motility**	**Number of Score**				**Pearson’s Chi-Squared Test**	**Bonferroni Correction**	
**at 12 °C**	**NG**	**D**	**H**	**Total**	***p*** **-Value**	**Adjusted** ***p*****-Value**	
B+H−	317	153	970	1440	*p* ≈ 0.0082	NG vs. D	0.158
B+H+	319	111	1010	1440		NG vs. M	1.000
Total	636	264	1980	2880		D vs. M	0.0063

## Data Availability

The genomes of the isolates are available on the database https://bigsdb.pasteur.fr/yersinia (accessed on 30 January 2026).

## Supplementary Materials

The following supporting information can be downloaded at: https://www.mdpi.com/article/10.3390/pathogens15050512/s1.

## Abbreviations

BT: biotype; cgMLST: core genome multi locus sequence typing; AD: allelic distance; BC: benzalkonium chloride; DDAC: Didecyl Dimethyl Ammonium Chloride; AMPD: N-(3-aminopropyl)-N-dodecylpropane-1,3-diamine; PA: peracetic acid; NaOCl: Sodium Hypochlorite.
